# TMPRSS2 Impacts Cytokine Expression in Murine Dendritic Cells

**DOI:** 10.3390/biomedicines11020419

**Published:** 2023-02-01

**Authors:** Sandra Gunne, Marie Schwerdtner, Marina Henke, Ann-Kathrin Schneider, Lucas Keutmann, Eva Böttcher-Friebertshäuser, Susanne Schiffmann

**Affiliations:** 1 Fraunhofer Institute for Translational Medicine and Pharmacology (ITMP), Theodor-Stern-Kai 7, 60596 Frankfurt am Main, Germany; 2 Institute of Virology, Philipps-University Marburg, 35043 Marburg, Germany

**Keywords:** TMPRSS2, dendritic cells, cytokine secretion, infection, TMPRSS2-knockout mouse

## Abstract

Background: The transmembrane protease serine 2 (TMPRSS2) proteolytically activates the envelope proteins of several viruses for viral entry via membrane fusion and is therefore an interesting and promising target for the development of broad-spectrum antivirals. However, the use of a host protein as a target may lead to potential side effects, especially on the immune system. We examined the effect of a genetic deletion of *TMPRSS2* on dendritic cells. Methods: Bone marrow cells from wild-type (WT) and TMPRSS2-deficient mice (TMPRSS2^−/−^) were differentiated to plasmacytoid dendritic cells (pDCs) and classical DCs (cDCs) and activated with various toll-like receptor (TLR) agonists. We analyzed the released cytokines and the mRNA expression of chemokine receptors, TLR7, TLR9, IRF7 and TCF4 stimulation. Results: In cDCs, the lack of TMPRSS2 led to an increase in IL12 and IFNγ in TLR7/8 agonist resiquimod or TLR 9 agonist ODN 1668-activated cells. Only IL-10 was reduced in TMPRSS2^−/−^ cells in comparison to WT cells activated with ODN 1668. In resiquimod-activated pDCs, the lack of TMPRSS2 led to a decrease in IL-6, IL-10 and INFγ. ODN 1668 activation led to a reduction in IFNα. The effect on receptor expression in pDCs and cDCs was low. Conclusion: The effect of TMPRSS2 on pDCS and cDCs depends on the activated TLR, and TMPRSS2 seems to affect cytokine release differently in pDCs and cDCs. In cDCs, TMPRSS2 seems to suppress cytokine release, whereas in pDCS TMPRSS2 possibly mediates cytokine release.

## 1. Introduction

The transmembrane protease serine 2 (TMPRSS2) has been shown to play an important role in the entry of respiratory viruses like influenza A virus (IAV) and various coronaviruses (CoV) by proteolytic activation of their envelope proteins for membrane fusion [1,2]. The recent severe acute respiratory syndrome coronavirus 2 (SARS-CoV-2) pandemic led to increased research on TMPRSS2 function and location. Nevertheless, much is still unknown about the physiological function of TMPRSS2. Its deficiency does not show a discernible phenotype in mice [3], while its roles in prostate cancer progression and virus infection have been described [4,5,6]. TMPRSS2 is mainly expressed in epithelial cells of the respiratory, gastrointestinal, and urogenital tract, with high expression levels in the prostate and colon. In the literature, detection of TMPRSS2 in immune cells is inconsistent. At least in monocytes, macrophages and dendritic cells (DC), low expression levels have been reported [7,8]. Moreover, it seems that TMPRSS2 expression levels change under disease condition [5,7,9]. While in some cancer types, like prostate cancer, TMPRSS2 is increased within the cancer tissue and correlates with poor survival rates, TMPRSS2 expression is decreased in lung cancer tissue, which correlates with poor outcomes. Some studies suggest a liaison between prostate cancer and COVID-19 and postulate a higher susceptibility of men due to TMPRSS2 regulation by androgens (summarized in [10]). Furthermore, during SARS-CoV-2 infection, TMPRSS2 expression significantly decreases [9], while pre-existing conditions, like diabetes, obesity, Barrett’s esophagus or autoimmune disease, show higher TMPRSS2 expression and are associated with a more severe course of SARS-CoV-2 [11]. Recent studies have found single nucleotide polymorphisms (SNPs) in the TMPRSS2 gene that are associated with higher TMPRSS2 expression and found to be a risk factor for severe IAV and SARS-CoV-2 infections in clinical studies [12,13,14]. Interestingly, *TMPRSS2* mRNA expression is increased by bacterial flagellin in human airway epithelial cells, suggesting that TMPRSS2 has a function in antimicrobial host response (Schwerdtner and Skalik et al., manuscript submitted). Furthermore, TMPRSS2 knockout mice showed a reduced/delayed immune response towards the immunostimulant poly I:C that resembles a double-stranded RNA and simulates viral infections [15]. Altogether, that might hint towards an immunomodulatory function of TMPRSS2.

DCs are a heterogeneous group of immune cells that bridge the innate and the adaptive immune system. Plasmacytoid dendritic cells (pDC) are a main source of type I interferon which recruits activated macrophages and therefore display a high anti-viral activity and are also involved in immune tolerance. Conventional/classical, also known as myeloid derived, dendritic cells (cDC) are antigen (Ag) processing and presenting cells and thereby interact with lymphocytes like T- and B-cells. Upon viral infection, infected cells undergo cytolyses and release pathogen-associated molecular patterns (PAMPs) and damage-associated molecular patterns (DAMPs) which attract DCs. cDC sense PAMPs and DAMPs through ligation with pattern recognition receptors (PRR) and cross-present antigens via major histocompatibility complexes (MHC) and release mainly IL-12 to activate T-cells and promote T helper (Th)-1 cells and natural killer cell responses. pDCs mainly sense single-stranded RNA and unmethylated CpG sequences in double-stranded DNA via their key endosomal pattern recognition receptors, the toll-like receptor 7 (TLR7) and TLR9, and respond with the release of type I interferon, especially interferon (IFN) α. In SARS-CoV-2 infections, it has been shown that patients display a reduced number of DCs and that especially the production of IFNα is impaired, even months after infection (summarized in [16]). Infections with pathogenic bacteria can be recognized by the immune system, either via unmethylated CpG sequences and TLR9 or by PAMPS on the bacterial surface, like lipopolysaccharide (LPS) or bacterial flagella, composed of the protein flagellin. The main recognition site for flagellin is the TLR5, which signals through myeloid differentiation primary response 88 (MyD88) and nuclear factor kappa-light-chain-enhancer of activated B-cells (NF-κB) to induce cytokine production.

This study was conducted to analyze the influence of TMPRSS2 on the function of dendritic cells. Since DCs have important roles during pathogen invasion, their relevant response should be identified before targeting TMPRSS2 for anti-viral treatment.

## 2. Materials and Methods

### 2.1. Animals

TMPRSS2-deficient C57BL/6J mice were kindly provided by Klaus Schughart and Luka Cicin-Sain (Helmholtz Centre for Infection Research, Braunschweig) and have been described previously [3,17]. Exons 10–13 were deleted by homologue recombination with a targeting vector containing a neomycin resistance gene as selection marker [3]. The truncated TMPRSS2 variant is enzymatically inactive. Homozygous TMPRSS2-knockout mice (TMPRSS2^−/−^) were bred and housed in the animal facility of the Philipps University of Marburg. C57BL/6J WT mice to be used as controls were purchased from Charles River Labs. The genotypes of mice were confirmed by genotyping (Figure S1, Supplementary information). All animal experiments were in accordance with the regulations of the Gesellschaft für Versuchstierkunde/Society of Laboratory Animal Science (GV-SOLAS) and approved by the local Ethics Committee for Animal Research in Darmstadt (approval number F152/2000) and the Commission on Animal Protection and Experimentation at the Philipps University of Marburg. Mice were housed in groups of 2–4 animals in GM500 Tecniplast cages with a Smartflow IVC air handling unit (Tecniplast Deutschland GmbH, Hohenpeißenberg, Germany) and were allowed to acclimatize for at least 7 days. Fourteen female C57BL\6 wild-type and 13 female TMPRSS2^−/−^ mice were taken for bone marrow extraction.

### 2.2. Isolation of Monocytes from Mouse Bone Marrow, Differentiation to Dendritic Cells and Treatment

Bone marrow cells were isolated from the femur and tibia of WT and TMPRSS2^−/−^ mice based on the protocol of Lui and Quan 2015 [18]. Briefly, mice were euthanized by isoflurane and cervical dislocation; hind limps were removed and freed from surrounding muscles and tissue. Bones were cut at both ends, the bone marrow flushed out and filtered through a 70 μm nylon cell strainer with ice-cold Hank’s Balanced Salt Solution (HBSS). After centrifugation, the cell pellet was resuspended in erythrocyte lysis buffer (0.16 M NH_4_Cl, 10 mM KHCO_3_, and 0.13 mM EDTA, in sterile H_2_O). The reaction was stopped after 2 min with PBS and the suspensions centrifuged. The cell pellet was resuspended in PBS and cell number was quantified using MACSQuant^®^ Analyzer 10 flow cytometer (Miltenyi Biotec, Bergisch Gladbach, Germany). Aliquots of 1.2 to 2 × 10^6^ cells were seeded onto six-well plates, either in Opti-MEM + 1% penicillin/ streptomycin, 1% fetal calf serum (FCS), 0.0008% β-mercaptoethanol and 200 ng/mL FLT3-ligand (PreproTech, Hamburg, Germany) for pDC differentiation or, with RPMI, 1% penicillin/streptomycin, 10% FCS, 0.0008% β-mercaptoethanol, 150 U/mL GM-CSF (PeproTech, Hamburg, Germany), and 75 U/mL murine IL-4 (Miltenyi Biotec, Bergisch Gladbach, Germany) for cDC differentiation. Cells were incubated at 37 °C and 5% CO_2_ to differentiate to cDC and pDC for 7 and 9 days, respectively.

Afterwards, cells were stimulated using fresh medium (without differentiation factors) with 20 ng/mL flagellin (Enzo Life Sciences GmbH, Loerrach, Germany), 12.5 µg/mL poly (I:C), 1 µg/mL, resiquimod (both Sigma-Aldrich, Taufkirchen, Germany), 0.2 µM ODN 1668 (InvivoGen, Toulouse, France), or remained untreated for another 48 h.

### 2.3. Verification of Differentiation

Differentiation of pDC and cDC cells from murine bone marrow cells was verified by flowcytometric analysis of CD45R and CD11c targeted cells. Therefore, 0.1 × 10^6^ cells were incubated at 4 °C for 10 min with mouse FcR blocking reagent (Miltenyi Biotec, Bergisch Gladbach, Germany), followed by a 15 min incubation with CD45R-APCVio770 and CD11c-FITC antibodies. After washing with FACS buffer (0.5% BSA, 2 mM EDTA, PBS) and centrifugation for 5 min, 300× *g*, cell pellet was resuspended in 100 µL FACS buffer and analyzed at MACSQuant^®^ Analyzer 10 flow cytometer.

### 2.4. Analysis of Secreted Cytokines

To detect potential differences in cytokine secretion in TMPRSS2^−/−^ mice under different stimulation conditions, supernatants of the 48 h treated cDCs and pDCs were collected and analyzed using Cytometric Bead Array (CBA; mouse; BD Bioscience, Heidelberg, Germany). IL-4, IL-6, IL-8, IL-10, IL-12 and IFN-γ were tested according to the assay manual and measured using the MACSQuant^®^ Analyzer 10 flow cytometer.

Furthermore, an IFNα ELISA (VeriKine Mouse Interferon alpha ELISA kit; BIOZOL Diagnostica, Eching, Germany) was used to analyze the IFNα secretion of pDCs, according to the manufacturer’s manual.

### 2.5. RNA Isolation, RT-PCR Analysis and qRT-PCR

For the determination of *TMPRSS2*-mRNA expression in different mouse tissues and immune cells lung, spleen and bone marrow (BM) from the same wild-type C57BL/6J mouse were used. cDCs and pDCs were derived from BM cells as described above. MΦ were derived from BM cells by differentiation using muM-CSF. Total RNA was isolated from tissues and cells using the RNeasy Mini Kit (QIAGEN, Hilden, Germany) according to the manufacturer’s protocol. Reverse transcription-PCR (RT-PCR) was carried out with total RNA using the one-step RT-PCR kit (QIAGEN) according to the supplier’s protocol. RT-PCR products were resolved on a 0.8% agarose gel stained with ethidium bromide. Identity of RT-PCR products was confirmed by sequencing. This experiment was repeated once.

For the detection of differentially expressed cytokine receptors and transcriptions factors, RNA of pDC and cDC cells were isolated after 48 h stimulation. The RNeasy Mini Kit (Qiagen, Hilden, Germany) including DNase digestion was used. Afterwards, cDC and pDC RNA were reverse transcribed to cDNA using First Strand cDNA synthesis kit and ReverseAid First Strand cDNA kit (Thermo Fisher scientific, Dreieich, Germany), respectively. The 10 µL qPCR Mix consists of 10 ng cDNA, 0.5 µM forward Primer, 0.5 µM reverse Primer, 1× Eva Green QPCR Mix (5× Mix, Bio&Sell, Feucht, Germany) and was used in a monoplex format on a 96-well plate. For information on primers used, see Table 1. As reference genes, RPL13A and PPIA were chosen, because they showed stable results in mouse tissue [19,20]. qPCR program was started with 15 min activation at 95 °C, followed by 40 cycles of 15 denaturation at 95 °C and 30 s annealing at 60 °C for cDC and 40 cycles of 15 s denaturation at 95 °C, 20 s annealing at 60 °C and 20 s expansion at 72 °C for pDC on a CFX96 Thermal Cycler from Bio-Rad. Raw data were analyzed by 2^−ΔΔCt^ method using Microsoft Excel.

### 2.6. Statistics

For graphical presentation and statistical analysis, GraphPad Prism 8 (GraphPad Software Inc., San Diego, CA, USA) was used. Data were tested for normality using the Kolmogorov–Smirnov test, and mean fold changes of qRT-PCR data were log_10_ transformed to achieve normal distribution. For normal distributed data, two-way ANOVA with Šídák’s multiple comparison was used to analyze datasets. Cytokine analysis of pDC cells did not achieve normal distribution, therefore multiple Mann–Whitney tests were performed. A *p* value > 0.05 was considered significant.

## 3. Results

### 3.1. TMPRSS2 Is Expressed in Murine Dendritic Cells

**Figure 1:**
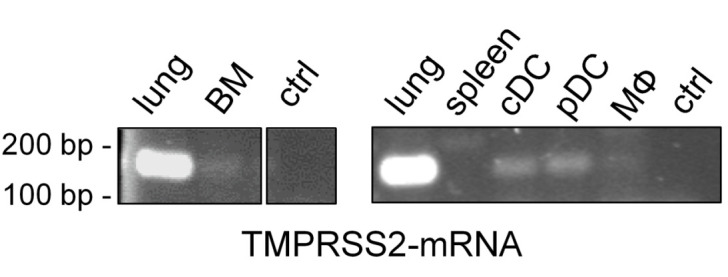
TMPRSS2 expression in murine immune cells and murine tissues. RT-PCR analysis of TMPRSS2-specific mRNA in bone marrow (BM) cells, spleen cells and from BM cells derived classical dendritic cells (cDCs), plasmacytoid DCs (pDCs) and macrophages (MΦ). Lung tissue and a no template control (ctrl) were used as positive and negative control, respectively.

Since the expression of TMPRSS2 in immune cells is controversial in the literature, an analysis of TMPRSS2 expression in murine bone marrow (BM) cells, DCs and macrophages (MΦ) using RT-PCR was performed. Figure 1 shows a comparison of TMPRSS2-mRNA expression in different cell types and tissues. Lung epithelial cells served as positive control and contained high amounts of TMPRSS2-mRNA, while spleen cells showed no expression. In BM cells, little TMPRSS2-mRNA was detected. Further differentiation of BM cells to cDC, pDC or MΦ revealed that DCs exhibit TMPRSS2 expression, although at lower levels compared to lung epithelial cells. In MΦ negligible TMPRSS2-mRNA could be detected.

### 3.2. TMPRSS2 Regulates the Secretion of Proinflammatory Cytokines

**Figure 2:**
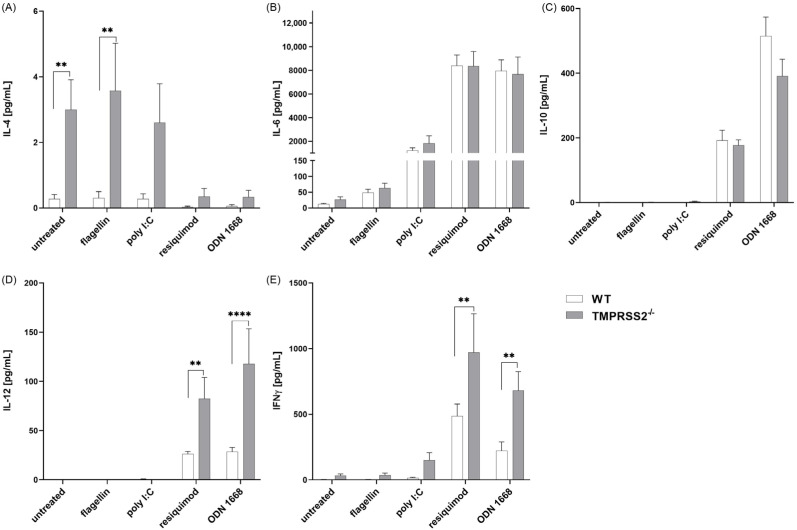
Cytokine levels in WT and TMPRSS2^−/−^ cDC. Murine bone marrow-derived cells were differentiated to cDC with GM-CSF and IL-4 for 7 days and stimulated with 20 ng/mL flagellin, 12.5 µg/mL poly I:C, 1 µg/mL resiquimod, or 0.2 µM ODN 1668 for another 48 h. Culture supernatants were analyzed using cytokine specific cytometric bead array. For statistical analysis, normality was tested using the Kolmogorov–Smirnov test and two-way ANOVA, with Šídák’s multiple comparison used. ** *p* < 0.01, **** *p* < 0.0001 indicate significant difference between WT and TMPRSS2^−/−^ cells. (**A**) IL-4 genotype effect: *p* > 0.0001, F(1,137) = 31.55; (**B**) IL-6 no genotype effect; (**C**) IL-10 no genotype effect, (ODN1668 *p* = 0.012); (**D**) IL-12 genotype effect: *p* = 0.0002, F(1,135) = 14.50; (**E**) IFNγ genotype effect: *p* = 0.0004, F(1,137) = 13.20. Animal numbers: WT = 14; TMPRSS2^−/−^ = 11–13.

To investigate whether functional TMPRSS2 has an influence on the immune response of DCs, the expression level of important receptors and factors, as well as levels of secreted cytokines, were determined in bone marrow-derived cDC from TMPRSS2-deficient mice (TMPRSS2^−/−^) and wild-type animals. TMPRSS2^−/−^ mice were generated by deletion of exon 10–13 through homologue recombination, resulting in expression of an truncated, inactive TMPRSS2 variant in TMPRSS2^−/−^ mice [3]. Successful differentiation was determined by CD11c expression on cDC cells after 7 days’ differentiation (Figure S2, Supplementary information). cDC were differentiated using GM-CSF/IL-4. Infection was simulated by 48 h stimulation with either poly I:C (viral), resiquimod (viral) or ODN 1668 (viral and bacterial). Resiquimod and ODN 1668 particularly led to an increased secretion of the cytokines IL-6, IL-10, IL-12 and IFNγ, whereas poly I:C led to a release of IL-6 and minor amounts of IFNγ (Figure 2). Flagellin-stimulated cells reacted comparably to the untreated cells. The cells reacted to resiquimod and ODN 1668 stimuli with high IL-6 secretion, but there was no difference in IL-6 release between WT and TMPRSS2^−/−^. Interestingly, the lack of TMPRSS2 led to an increase in the interdependent cytokines IL-12 and IFNγ in cDC cultures under resiquimod and ODN 1668 stimulation, which was significantly higher than in the WT. The release of IL-4 was significantly increased in TMPRSS2^−/−^ cDCs activated with poly I:C or flagellin as well as in unstimulated cells, although the amount was in the low picogram range. The anti-inflammatory cytokine IL-10 was secreted from TMPRSS2^−/−^ cDC cultures significantly less after ODN 1668 stimulation when compared to WT. These data showed that the lack of TMPRSS2 in cDC leads to an increase in IL-4, IL-12 and INFγ, depending on the activation stimulus. This indicates that TMPRSS2 act as a suppressor of cytokine release.

### 3.3. TMPRSS2 Expression Has Only Minor Effects on Chemokine Receptor Expression in cDC

**Figure 3:**
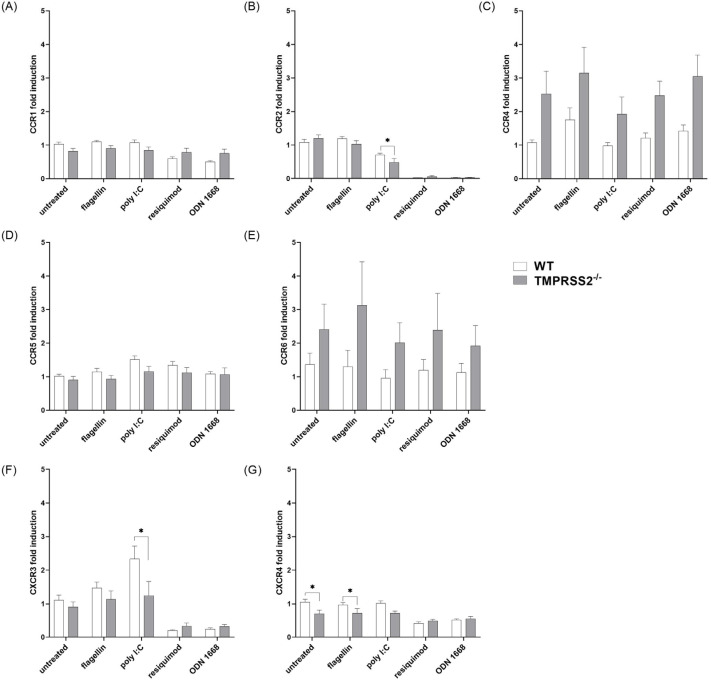
Expression levels of important cDC receptors. Murine bone marrow-derived cells were differentiated to cDC with GM-CSF and IL-4 for 7 days and stimulated with 20 ng/mL flagellin, 12.5 µg/mL poly I:C, 1 µg/mL resiquimod, or 0.2 µM ODN 1668 for another 48 h. Total RNA was isolated and analyzed by qRT-PCR. Expression levels were analyzed by the 2^−ΔΔCt^ method, normalized to the geometric mean of reference genes RPL13A and PPIA and to the untreated samples. For statistical analysis, two-way ANOVA with Šídák’s multiple comparison was used. * *p* < 0.05 indicate significant difference between WT and TMPRSS2^−/−^ cells within the same treatment. (**A**) CCR1 no genotype effect; (**B**) CCR2 no genotype effect (poly I:C *p* = 0.0104); (**C**) CCR4 genotype effect: *p* > 0.0001, F(1,118) = 17.10; (**D**) CCR5 genotype effect *p* = 0.003, F(1,93) = 9.321; (**E**) CCR6 genotype effect: *p* = 0.0213, F(1,93) = 5.490; (**F**) CXCR3 no genotype effect, (poly I:C *p* = 0.0453); (**G**) CXCR4 genotype effect: *p* = 0.0038; F(1,118) = 8.743. Animal numbers: WT = 14; TMPRSS2^−/−^ = 11–13.

Next, the mRNA expression levels in cDC of the receptors CCR1, CCR2, CCR4, CCR5, CCR6, CXCR3, and CXCR4 were analyzed by qRT-PCR (Figure 3). The lack of functional TMPRSS2 in cDC did only minorly influence the expression levels of CCR1, CCR2 and CCR5. There was a stimulant independent genotype difference in CCR4 and CCR6 expression, with increased levels in the TMPRSS2^−/−^ cells (Figure 3C,D). In contrast, CXCR3 and CXCR4 were decreasingly expressed in TMPRSS2^−/−^ cDC cultures compared to WT, especially for CXCR3 under poly I:C stimulation and for CXCR4 in untreated and flagellin treated cells, although the effect was minimal and therefore, probably physiologically irrelevant (Figure 3F,G). Interestingly, this effect was not present under resiquimod and ODN 1668 stimulation, where a genotype-independent reduced expression of CXCR3 and CXCR4 was detected. These data indicate that the lack of TMPRSS2 has only minor effects on chemokine receptor expression in cDCs, with the exception of CCR6, which is normally associated with immature DC, and CCR4, which is predominantly expressed on TH2 and Treg cells and associated with DC-T-cell binding.

### 3.4. TMPRSS2^−/−^ pDCs Show Lower Cytokine Secretion Levels after Resiquimod and ODN 1668 Stimulation Compared to WT

**Figure 4:**
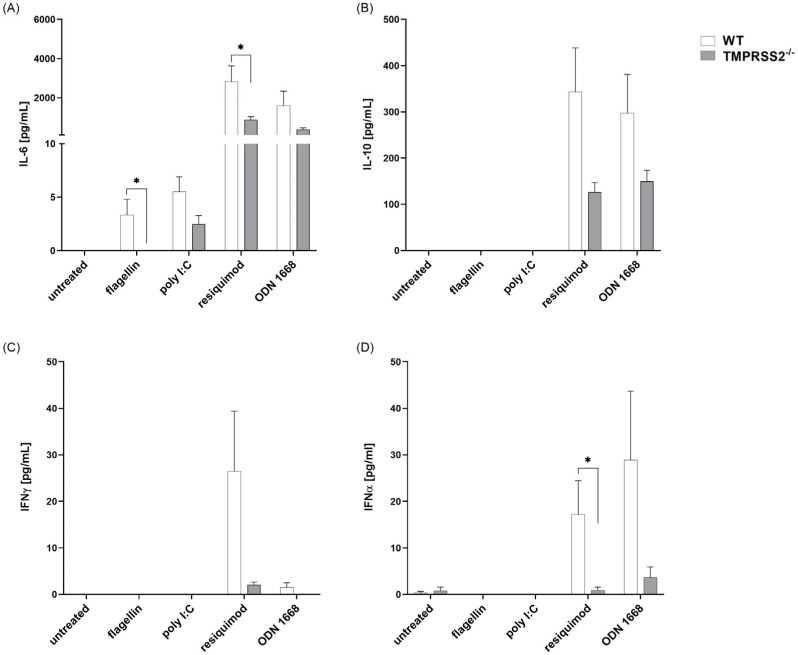
Cytokine levels in WT and TMPRSS2^−/−^ pDC. Murine bone marrow-derived cells were differentiated to pDC with FLT3-ligand for 9 days and stimulated with 20 ng/mL flagellin, 12.5 µg/mL poly I:C, 1 µg/mL resiquimod, and 0.2 µM ODN 1668 for another 48 h. (**A**) IL6, (**B**) IL-10, (**C**) IFNγ and (**D**) IFNα were detected in culture supernatants and analyzed using cytokine-specific cytometric bead array or ELISA (IFNα). For statistical analysis, multiple Mann–Whitney tests were used. * *p* < 0.05 indicate significant difference between WT and TMPRSS2^−/−^ cells. Animal numbers: WT = 7–8; TMPRSS2^−/−^ = 7–8.

pDCs were differentiated using FLT3-ligand and, as expected, the expression of CD45R was increased after the 9 days of differentiation. pDCs were stimulated with flagellin, poly I:C, resiquimod or ODN 1668 and secretion of IL-6, IL-10, IFNγ, as well as IFNα, which is mainly produced by pDCs, were determined in the supernatant (Figure 4). In pDCs, resiquimod induced the release of IL-6, IL-10, IFNγ and IFNα, whereas ODN 1668 induced the release of IL-6, IL-10 and IFNα. Poly I:C and flagellin only led to a minimal secretion of IL-6 and had no effect on the other cytokines. In untreated cells, only a minor release of IFNα was detected. Interestingly, a lack of TMPRSS2 in resiquimod-activated pDCs led to a reduced release of IL-6 and INFα, with the same tendency in IL-10 and IFNγ. In ODN 1668-activated pDCs, the lack of TMPRSS2 tend to lower the secretion of all measured cytokines, but these effects were not significant. These data indicate that, in pDCs, TMPRSS2 has an opposing action to that in cDC, since functional TMPRSS2 seems to mediate cytokine release.

### 3.5. TMPRSS2^−/−^ pDCs Show Lower Expression of Motility Relevant Receptors and Important Type I IFN Inducing Proteins

After stimulation, pDCs show elevated levels of TRF7 and IRF7, which are part of one important signaling cascade for IFNα induction, especially in the WT cells’ expression levels were elevated. When stimulated with resiquimod or ODN 1668, WT expression was increased around 100-fold and 75-fold compared to the untreated condition, respectively. Interestingly, IRF7 expression levels were also increased under flagellin (WT 21-fold; TMPRSS2^−/−^ 14-fold) and poly I:C treatment (WT 51-fold; TMPRSS2^−/−^ 28-fold), but this seemed not to be enough to induce detectable IFNα secretion. Furthermore, one receptor upstream of the signaling cascade of IRF7, the TLR9, showed increased expression with resiquimod and ODN 1668 treatment, but did not differ from expression in the presence of TMPRSS2.

**Figure 5:**
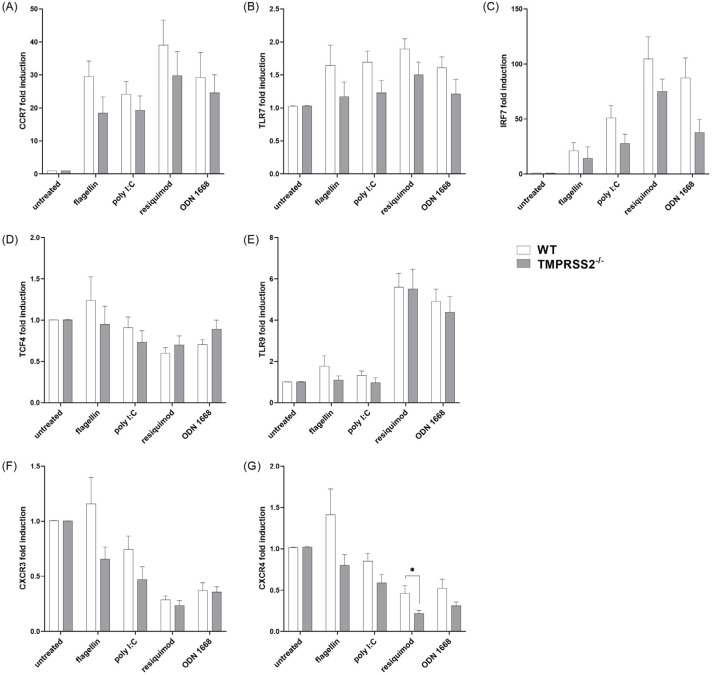
Expression levels of important pDC receptors and transcription factors. Murine bone marrow-derived cells were differentiated to pDC with FLT3-ligand for 9 days and stimulated with 20 ng/mL flagellin, 12.5 µg/mL poly I:C, 1 µg/mL resiquimod, and 0.2 µM ODN 1668 for another 48 h. Total RNA was isolated and analyzed by qRT-PCR. Expression levels were analyzed by the 2^−ΔΔCt^ method, normalized to the geometric mean of reference genes RPL13A and PPIA and to the untreated samples. For statistical analysis, fold changes were log_10_ transformed and two-way ANOVA with Šídák’s multiple comparison was used. * *p* < 0.05 indicate significant difference between WT and TMPRSS2^−/−^ cells. (**A**) CCR7 genotype effect: *p* = 0.0225, F(1,63) = 5.476 (**B**) TLR7 genotype effect: *p* = 0.0034, F(1,63) = 9.276; (**C**) IRF7 genotype effect: *p* = 0.004, F(1,63) = 8.939; (**D**) TCF4 no genotype effect; (**E**) TLR9 no genotype effect; (**F**) CXCR3 genotype effect: *p* = 0.0136, F(1,63) = 6.442; (**G**) CXCR4 genotype effect: *p* > 0.0001, F(1,63) = 17.45. Animal numbers: WT = 7–8; TMPRSS2^−/−^ = 7–8.

Similarly to the cDC cultures, the expression of the receptors CXCR3 and CXCR4, which are responsible for immature pDC movement, was reduced in pDCs under TLR7/TLR9 stimulating conditions (Figure 5F,G), whereas CCR7, the most important receptor for mature pDC migration, was elevated 20- to 40-fold in all stimulated conditions compared to the untreated condition; there was no difference between the genotypes.

## 4. Discussion

The serine protease TMPRSS2 has been reported to play a role in different diseases. It is therefore under consideration as a potential treatment target. Most studies focus on TMPRSS2 function in disease conditions, like cancer or viral infection. TMPRSS2 might play a role in pain experience, especially for cancer patients [21]. Several substrates have been suggested for TMPRSS2, like protease-activated receptor-2 (PAR-2), serine protease-like zymogens, or the single-chain precursor of hepatocyte growth factor (HGF) (summarized in [22]). However, since TMPRSS2 deficiency does not show a discernible phenotype in mice and it is known that many human proteases show functional redundancy, a physiological function still remains elusive. To shed more light on TMPRSS2 function, and especially the consequences of its absence, this study aims to examine the role of TMPRSS2 in the immune system on the example of dendritic cells.

Although DCs only express low levels of TMPRSS2 compared to epithelial cells of the respiratory, gastrointestinal, or urogenital tract, the present study shows that a functional knockout of TMPRSS2 leads to an altered cytokine secretion pattern under simulation of infection. To simulate infections, various TLR agonists were used. Poly I:C, resiquimod and ODN 1668 are agonists of TLR3, TLR7/8 or TLR9, respectively, which also detected viral nucleic acids and unmethylated CpG sequences [23]. Furthermore, bacterial infection was simulated using flagellin (agonist of TLR5). IL-6, IL-10 and IL-12 were analyzed due to their importance in communication of DCs with T and B-cells [24,25,26], while IL-4 and IFNγ are autocrine mediators for DC maturation [27,28]. We found that cDCs differentiated from bone marrow cells of TMPRSS2^−/−^ mice secrete significantly more IL-12 and IFNγ than cDC cultures from wild-type when stimulated with resiquimod or ODN 1668. This is associated with a helper T-cell (TH) 1 response, which activates macrophages and further proinflammatory cytokine secretion [29]. IL-4, responsible for TH2 differentiation of T helper cells, is predominantly expressed in TMPRSS2^−/−^ cDCs under conditions of unstimulated, flagellin or poly I:C stimulation. Furthermore, less IL-10 was secreted by the TMPRSS2^−/−^ cDCs under resiquimod or ODN 1668 induction. We therefore observed a more proinflammatory reaction to infection simulation with RNA or unmethylated CpG sequences in cDC cultures in the absence of functional TMPRSS2. This is clinically relevant, since it has been found that, in COVID-19 patients, an imbalance in TH2/TH1 response is associated with higher mortality [30].

In contrast, pDC cultures differentiated from TMPRSS2^−/−^ mouse bone marrow cells showed reduced secretion of all analyzed cytokines compared to the WT pDCs. This hints towards dysfunction of pDCs in the absence of functional TMPRSS2. However, since all measured cytokines are reduced in the TMPRSS2^−/−^ cells, this could also be an effect of less viable cells in the TMPRSS2^−/−^ pDC cultures after the 9 days of differentiation. A reduced viability might indicate differentiation problems of pDCs lacking functional TMPRSS2. Even though TMPRSS2^−/−^ mice showed no obvious phenotype change [3,15], a detailed analysis of the immune cell status has yet not been described. It has been shown that TMPRSS2^−/−^ mice not only are resistant towards IAV and CoV infection, including SARS-CoV and Middle East respiratory syndrome (MERS), but also show different immunopathology and reduced inflammatory lesions [15,31]. Therefore, it is possible that TMPRSS2 function is not mandatory for normal mouse development and in vivo cell viability, but could play a role in challenging situations, like infections or just during in vitro culturing and differentiation of cells.

The impaired IFNα production seen in pDCs without functional TMPRSS2 also has clinical relevance, since, in SARS-CoV-2 infection, it has been shown that DCs are impaired and IFNα production is reduced [16]. Furthermore, it has been shown that TMPRSS2 is reduced in COVID-19 patients [9]. Since the loss of TMPRSS2 has also been shown to lead to a delayed and reduced production of IFNα and that, in our study, IFNα production was impaired in pDCs without functional TMPRSS2, there might be a common mechanism. We observed reduced expression of the IFNα transcription factor IRF7 and one of its upstream signaling receptors, the TLR7, in TMPRSS2^−/−^ pDCs, indicating that the detection site for single-stranded RNA is reduced. Moreover, the reduction of TMPRSS2 seen in SARS-CoV-2 infection, together with our findings that cDCs lacking TMPRSS2 react with more proinflammatory cytokine secretion, might explain the tissue damage in the lungs of severe diseased patients [9].

A limitation of this study might be that we cannot exclude the possibility that the observed effects are not solely related to cDCs or pDCs in our differentiated cultures, since FLT-3 ligand can also be used to differentiate cDCs from bone marrow cells [32] and cultures using GM-CSF can also include monocyte-derived macrophages [33,34]. Nevertheless, we did see an enrichment of CD11c and CD45R in GM-CSF- and FLT-3 ligand-stimulated cultures, respectively (Figure S2, Supplementary information). Furthermore, the chosen stimulation times of 48 h with the TLR agonist might not be the optimal time point for the analysis of protein expressions on the RNA level (qRT-PCR) and, therefore, only minor effects were detected. Moreover, since DCs secrete a variety of cytokines, there might be further interesting and potentially TMPRSS2-influenced cytokines. To find these, a RNAseq could be performed.

Since TMPRSS2 downregulation has been shown to reduce virus titers and infectivity in vitro and in vivo, TMPRSS2 is thought to be a good target for the treatment of infections with viruses that enter the host using this protease. Unfortunately, in clinical trials with serine protease inhibitors, like camostat, time to clinical improvement in COVID-19 patients was not affected [35,36]. This could likely be an effect of TMPRSS2 downregulation in DCs, as the altered immune response we observed in our study suggests.

## 5. Conclusions

In conclusion, our study indicates that TMPRSS2, although only slightly expressed in dendritic cells, alters the immune response during infection simulation. TMPRSS2 might therefore not be necessary for the physiological function, but may be important under challenging conditions. Moreover, the effect of TMPRSS2 on pDCs and cDCs depends on the type of activated TLR, and TMPRSS2 seems to affect cytokine release differently in pDCs and cDCs. In cDCs, TMPRSS2 seems to suppress cytokine release, whereas in pDCs TMPRSS2 seems to mediate cytokine release.

## Figures and Tables

**Table 1:** Primer list.

Primer Name	Forward (5′-3′)	Reverse (5′-3′)
RPL13A (reference)	TGCCCCACAAGACCAAGAGAG	TGAGAGCAGCAGGGACCACC
PPIA (reference)	GCTGGACCAAACACAAACGG	GCCATTCCTGGACCCAAAAC
CCR1	CCATCATCATACAGGAAGCCAAG	TCTGTGAAATCTGAAATCTCCATCC
CCR2	ATCCACGGCATACTATCAACATCTC	GACAAGGCTCACCATCATCGTAG
CCR4	ATCACTTTCAGAAGAGCAAGGCAG	CAGTTTCATCCTGGGTGGTGTC
CCR5	CAGAGGAGGTGAGACATCCGTTC	TTTTGGCAGGGTGCTGACATAC
CCR6	GTCCTGGGTGCTTAGAGACT	CAAGGGCATGACTCCTTTCCA
CCR7	CCAGCAAGCAGCTCAACATT	GCCGATGAAGGCATACAAGA
CXCR3	TGAGGTTAGTGAACGTCAAGTGCT	CCCCATAATCGTAGGGAGAGGT
CXCR4	CACGGCTGTAGAGCGAGTGTTG	ATGAAGTAGATGGTGGGCAGGAAG
TLR7	TTCTCTTCAGCATGTGCCCC	GACATACCCCTTGACACGCA
TLR9	GAGAAGCAACCCTCTGCACT	ATCGAACACCACGAAGGCAT
IRF7	TCCAGTTGATCCGCATAAGGT	CTTCCCTATTTTCCGTGGCTG
TCL4 (B2-2)	CCAGGAACCCTTTCGCCCACCAAAC	TGCTGGCTGCTGGCTTGGAGGAA
TMPRSS2	AAGGGATACCAGACCAGAGC	GCGAACCCCGCATTCTATAC

## Data Availability

The data that support the findings of this study are available from the corresponding author upon reasonable request.

## Supplementary Materials

The following supporting information can be downloaded at: https://www.mdpi.com/article/10.3390/biomedicines11020419/s1, Figure S1: TMPRSS2^−/−^ mice genotyping; Figure S2: Differentiation profile of cDCs and pDCs.
