# Supplementary material for: TMPRSS2 Impacts Cytokine Expression in Murine Dendritic Cells
Source: Biomedicines. 2023 Feb 1;11(2):419. doi: 10.3390/biomedicines11020419 (PMC9952936; doi:10.3390/biomedicines11020419)
Supplement: Supplementary file 1 [file biomedicines-11-00419-s001.zip › biomedicines-2134170-supplementary.pdf]

Supplementary material:

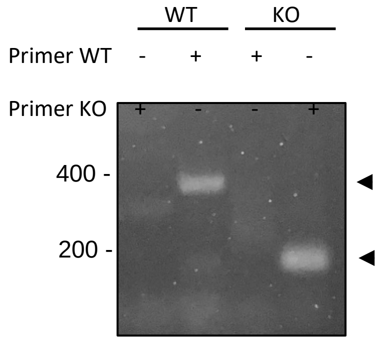

Figure S1 TMPRSS2$^{-/-}$ mice genotyping

TMPRSS2$^{-/-}$ mice were generated by deletion of exons 10–13 through homologue recombination with a targeting vector containing a neomycin resistance gene as selection marker (Kim et al., 2006; [3] main publication). The truncated TMPRSS2 variant is enzymatically inactive. Genotyping by PCR analysis was carried out using primers WT-for and WT-rev to amplify a 385 bp allele from the wild-type animals (primers described in [3] main publication) and primers KO-for and KO-rev to amplify 180 bps of the neomycin gene.

WT-for 5’ ACCTGGAGTATACGGGAACGTGA 3’
WT-rev 5’ GTGAGTGGGTGAAGGTTGGGTAG 3’
KO-for 5’ CTACGCGTCAATTGATGCATCCC 3’
KO-rev 5’ CTGCTAAAGCGCATGCTCCAGAC 3’

References

[3] Kim, T.S.; Heinlein, C.; Hackman, R.C.; Nelson, P.S. Phenotypic analysis of mice lacking the Tmprss2-encoded protease. *Mol. Cell. Biol.* **2006**, *26*, 965–975. https://doi.org/10.1128/MCB.26.3.965-975.2006.

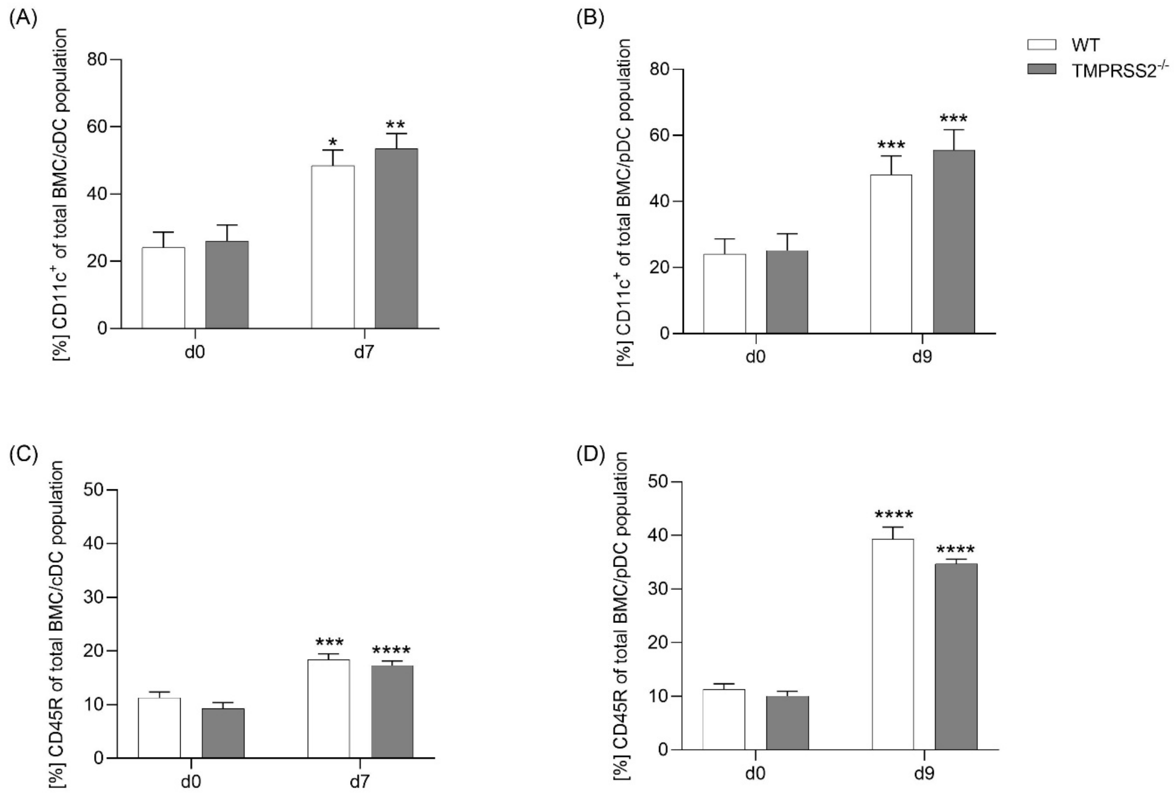

Figure S2 Differentiation profile of cDCs and pDCs

Differentiation of pDC and cDC cells from murine bone marrow cells was verified by flow cytometric analysis of CD45R and CD11c targeted cells. Therefore, 0.1x10^6 cells were incubated at 4 °C for 10 min with mouse FcR blocking reagent (Miltenyi Biotec, Bergisch Gladbach, Germany), followed by a 15 min incubation with CD45R-APCVio770 and CD11c-FITC antibodies. After washing with FACS buffer (0.5 % BSA, 2 mM EDTA, PBS) and centrifugation for 5 min, 300 xg, cell pellet was resuspended in 100 µl FACS buffer and analyzed at MACSQuant® Analyzer 10 flow cytometer. *p<0.05, **p<0.01, ***p<0.001 and ****p<0.0001 showed significant differences between day7/day9 and day 0.
